# Renoprotective effect of vildagliptin following hepatic ischemia/reperfusion injury

**DOI:** 10.1080/0886022X.2020.1729189

**Published:** 2020-02-26

**Authors:** Iman O. Sherif, Alaa A. Alshaalan, Nora H. Al-Shaalan

**Affiliations:** a Emergency Hospital, Faculty of Medicine, Mansoura University, Mansoura, Egypt; b College of Pharmacy, King Saud University, Riyadh, Saudi Arabia; c Chemistry Department, College of Science, Princess Nourah bint Abdulrahman University, Riyadh, Saudi Arabia

**Keywords:** Hepatic ischemia/reperfusion, renal injury, vildagliptin, TGF-β, α-SMA

## Abstract

Remote renal injury is a drastic consequence of hepatic ischemia/reperfusion (IR) injury. Vildagliptin (V) is a dipeptidyl peptidase-4 inhibitor that has a hepatorenal protective effect against models of liver and renal IR. This research was done to explore the protective role of vildagliptin against renal injury following hepatic IR injury as well as the possible involvement of transforming growth factor-beta (TGF-β)/Smad/alpha-smooth muscle actin (α-SMA) expressions in the pathophysiological mechanism of the remote renal injury. Three groups of male Wistar rats were organized into: sham group, IR group, and V + IR group in which 10 mg/kg/day of vildagliptin was pretreated for 10 days intraperitoneally. Blood in addition to renal and hepatic tissue samples was used for biochemical and histopathological studies. Hepatic IR induced a marked increase in serum creatinine, blood urea nitrogen, liver enzymes, renal nitric oxide, malondialdehyde, tumor necrosis factor-alpha levels with a marked upregulation of renal mRNA expressions of TGF-β, Smad2, Smad3, and α-SMA in addition to a marked decline in renal catalase content comparing to the sham group. Abnormal histopathological findings of hepatic and renal injury were detected in the IR group. Vildagliptin significantly improved these biochemical markers as well as the histopathological changes. The upregulation of renal TGF-β/Smad/α-SMA mRNA expressions was involved for the first time in the pathogenesis of the renal injury following hepatic IR and vildagliptin ameliorated this renal injury through blocking these expressions.

## Introduction

Intraoperative blood loss has been encountered in liver surgeries including resection and transplantation. It is a life-threatening condition that could be managed temporarily by clamping the portal triad and this may result inadvertently to a process of ischemia following by a reperfusion phase which exacerbates the hepatic injury depending on the duration of the two phases [1,2].

Prolonged liver ischemia followed by reperfusion stimulates the induction of pro-inflammatory cytokines as well as the reactive oxygen species (ROS) leading to hepatic cell as well as remote organ injury accompanied with high morbidity and mortality [3,4]. The injury induced by liver ischemia/reperfusion (I/R) in lung and kidney has been reported [5,6]. However, the exact mechanism underlying the renal injury induced by liver I/R has not been fully determined.

Till now, researchers are searching for suitable pharmacological interventions to minimize the kidney injury produced after hepatic I/R [5]. Dipeptidyl peptidase-4 (DPP-4) inhibitors, including linagliptin, sitagliptin, and vildagliptin (V), are class of hypoglycemic agents used for treating type II diabetes through inhibition of glucagon-like peptide-1 (GLP-1) degradation [7]. Previous studies found that DPP-4 inhibitors like sitagliptin and vildagliptin could protect against tubular damage that occurred in renal I/R injury via suppression of oxidative stress, apoptosis as well as inflammatory mediators [8–10].

Recently, our research team reported the hepatoprotective effect of vildagliptin in the hepatic I/R injury [11]; however, there are still no findings in the literature showed the effect of vildagliptin against acute kidney injury induced by liver I/R. Therefore, this work was designed to elucidate the pathophysiological mechanism underlying the renal injury induced by hepatic I/R and the possible renoprotective effect of using vildagliptin.

## Materials and methods

### Animals

Male Wistar rats with weights ranging 250–300 g were kept in cages exposing to certain conditions (12 h light/dark cycle, 20–25 °C) and they were allowed free access to standard chow and tap water *ad libitum*. The research protocol was approved from the Animal Ethics Committee of the Nile Center for Experimental Researches, Egypt. The handling of animals was in accordance with the guide for the Care and Use of Laboratory Animals (NIH publication no. 85-23, revised 2011).

### Experimental design

Thirty male Wistar rats were distributed into three groups (*n* = 10) as following:

- *Sham group*: Subjected to surgery without clamping the portal triad and received 5 ml/kg normal saline.
- *I/R (IR) group*: Subjected to surgery with clamping the portal triad and received normal saline.
- *Vildagliptin + I/R (V + IR) group*: Subjected to surgery with clamping the portal triad and pretreated with 10 mg/kg/day vildagliptin (V, Galvus, Novartis Pharma Stein AG, Stein, Switzerland) intraperitoneally injected for 10 days, and 15 min before ischemia at the time of surgery. The dose, duration, and route of administration of vildagliptin were selected based on the reported protective effect of vildagliptin in different animal models [10–13].

### Model of ischemia/reperfusion injury

**Figure 1.**
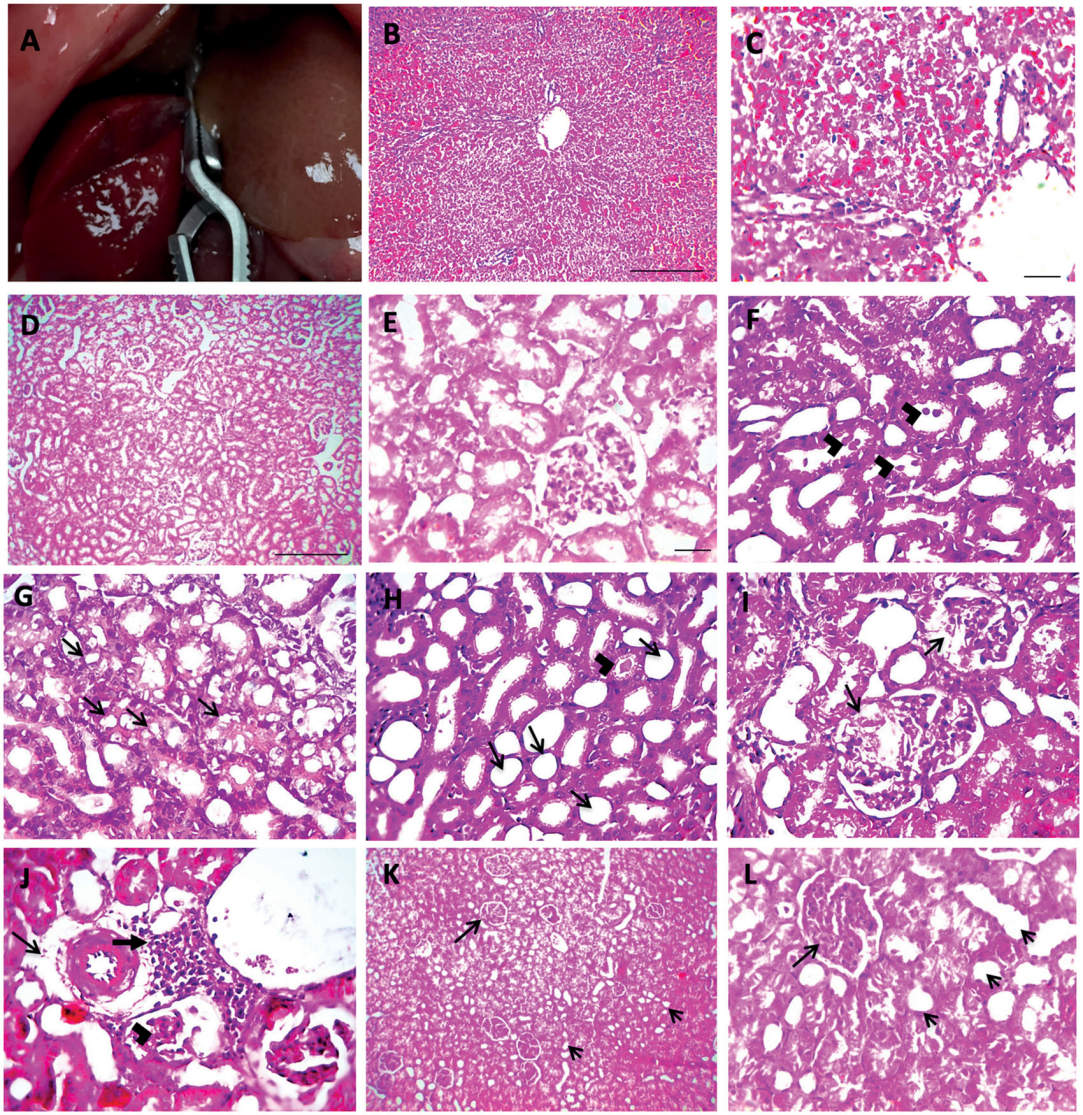
Hepatic ischemia in rat liver (A). Histopathology of liver in hepatic IR group with hematoxylin and eosin (H&E). Low ×100, scale bar = 100 µm (B) and high ×200, scale bar = 50 µm (C) magnification power showed severe sinusoidal congestion and necrosis with hydropic degeneration in hepatocytes. Histopathological pictures of renal tissues stained with H&E from experimental groups (D–L). Kidney showing normal histological picture in sham group (D, E). Kidney sections from IR group showing tubular dilation with presence of hyaline bodies in lumen of tubules (arrowheads) (F), vacuolar degeneration in tubular epithelium (arrows) (G), tubular dilation with loss of brush border (arrows) and tubular cast (arrowhead) (H), swollen Bowman’s capsule with eosinophilic proteinaceous material (arrows) (I), few perivascular edema (thin arrow) with mononuclear cells infiltration (thick arrow) and glomerular shrinkage (arrowheads) (J). Glomerular hypercellularity (long arrow) with loss of brush border from tubular epithelium (short arrows) (K, L) was observed in V + IR group (D, K scale bar = 100 µm, ×100; E scale bar = 50 µm, ×200; F, G, H, I, J scale bar = 50 µm, ×400).

The model of renal injury after liver I/R injury was done as mentioned before in previous research [5,14] in which the liver I/R injury was first implemented. Briefly, rats in our experiment were anesthetized and subjected to a midline laparotomy under aseptic condition. After that, the portal triad was identified and clamped by a Bulldog clamp for 45 min to achieve liver ischemia and this was confirmed by the pale color of the median and left liver lobes (Figure 1(A)). After the period of the ischemia, the clamp was removed and reperfusion for 3 h was done which was confirmed by the return of the reddish-brown color of the liver then removal of kidneys and liver was carried out.

### Blood and tissue sample collections

At the end of reperfusion, samples of blood were collected and centrifuged at 3000 rpm for 5 min after that, the serum was kept at –20 °C for renal and liver function determination. Moreover, parts of the kidney tissues were removed and immersed immediately in liquid nitrogen and stored at –80 °C for further investigations while other parts of the kidney and liver were immersed in 10% formalin for histopathology.

### Evaluation of the renal and liver function tests

The assessment of the renal function was done through the measurement of serum creatinine (Cr) (cat. no. CR 1250), and blood urea nitrogen (BUN) (cat. no. UR 2110) while liver function was evaluated via the measurement of serum aspartate transaminase (AST), and alanine transaminase (ALT) (cat. no. AT 1034) using kits purchased from Bio-Diagnostic Company (Giza, Egypt).

### Histopathological examination

Renal and hepatic tissues were fixed in formalin solution (10%), processed, and embedded in paraffin. Serial coronal sections of 5-μm thick are obtained by a microtome and stained with hematoxylin and eosin (H&E) for light microscopy. The histopathological assessment was performed in a blinded way by a pathologist.

### Estimation of renal oxidative stress markers

The malondialdehyde (MDA) (cat. no. MD 2529), catalase (cat. no. CA 2517), and nitric oxide (NO) (cat. no. NO 2533) were detected in the kidney tissue homogenate after preparation as followed: a piece of the renal tissue was ice-cooled, after that, homogenized in 10-fold phosphate buffer (pH 7.4) and at last centrifuged at 600×*g* for 10 min at 4 °C. Oxidative stress markers were determined colorimetrically using kits obtained from Bio-Diagnostic Company (Giza, Egypt) according to the manufacturers’ instructions.

### ELISA technique for inflammatory marker determination

To assess the inflammation, the renal level of tumor necrosis factor-alpha (TNF-α) was measured using a rat TNF-α ELISA kit (Cloud Clone Corp, Houston, TX, cat. no. SEA133Ra) by following the manufacturers’ instructions.

### Real-time quantitative-polymerase chain reaction (qPCR) for transforming growth factor-β (TGF-β), smad2, smad3, and α-smooth muscle actin (α-SMA) gene expressions determination

Detection of renal tissue of TGF-β, smad2, smad3, and α-SMA mRNA expressions was performed by qPCR. The total RNA was extracted from renal tissues, reverse transcribed into complementary DNA (cDNA), and amplified by qPCR using RNeasy Mini Kit (QIAGEN, Valencia, CA, cat. no. 74104), High-Capacity cDNA Reverse Transcription kit (Applied Biosystems, Foster City, CA, cat. no. 4368814), and SYBR Green PCR Master Mix Kit (Applied Biosystems, Foster City, CA, cat. no. 4309155), respectively by following the manufacturer’s instructions. The ratio of A260/A280 was used to analyze the quantity and quality of the extracted RNA while the gel electrophoresis on a 1% agarose gel was used to study the RNA integrity. Relative expressions of genes were calculated by the ΔΔCt method. All values were normalized to the GAPDH genes. The primers sequences of the genes are presented in Table 1.

**Table 1.** Primer sequences of the studied genes.

Target gene	Primer sequence: 5′ → 3′
TGF-β	F: TGCGCCTGCAGAGATTCAAG
	R: AGGTAACGCCAGGAATTGTTGCTA
Smad2	F: GCCCCAACTGTAACCAGAGA
	R: GCCAGAAGAGCAGCAAATTC
Smad3	F: GGCTTTGAGGCTGTCTACCA
	R: GGTGCTGGTCACTGTCTGTC
α-SMA	F: GTTTGAGACCTTCAATGTCCC
	R: CGATCTCACGCTCAGCAGTGA
GAPDH	F: CACCCTGTTGCTGTAGCCATATTC
	R: GACATCAAGAAGGTGGTGAAGCAG

### Statistical analysis

Data were expressed as mean ± SD. The differences between groups were assessed by the one-way analysis of variance (ANOVA) followed by a *post hoc* Bonferroni test. Statistical significance was considered when the *p* value is <.05. Statistical analysis was carried out by using the computer software SPSS version 20 (Chicago, IL).

## Results

### Effect on renal and liver function

In Table 2, hepatic I/R produced a marked increase in serum Cr, BUN, AST, and ALT compared to the sham group. Pretreatment of vildagliptin for 10 days exhibited a notable reduction in serum Cr and BUN levels as well as the levels of AST and ALT when compared with the IR group (*p*<.001).

**Table 2.** Effect of liver ischemia/reperfusion (IR) alone or in combination with vildagliptin (V, 10 mg/kg, intraperitoneally) administration for 10 days on the serum levels of creatinine (Cr), blood urea nitrogen (BUN), aspartate transaminase (AST), and alanine transaminase (ALT) in all experimental animals.

Group	Sham	IR	V + IR
Cr (mg/dl)	0.65 ± 0.13	1.33 ± 0.18*	0.85 ± 0.09^##^
BUN (mg/dl)	36.54 ± 1.46	62.86 ± 5.5*	45.54 ± 2.3**^,^^#^
AST (U/l)	51 ± 3.16	117.4 ± 6.9*	73.5 ± 4.2*^,^^#^
ALT (U/l)	29.5 ± 2.87	99.4 ± 10.33*	48.2 ± 5.02**^,^^#^

Results were expressed as mean ± SD.

^*^ *p*<.001 compared to sham group.

^**^ *p*<.05 compared to sham group.

^#^ *p*<.001 compared to IR group.

^##^ *p*<.05 compared to IR group.

### Effect on liver histopathology

To confirm the injury in liver tissue after hepatic IR, liver sections examined following hepatic IR. Liver tissue in the IR group showed severe sinusoidal congestion, necrosis with hydropic degeneration in hepatocytes throughout hepatic lobules (Figure 1(B,C)).

### Effect on renal histopathological examination

Figure 1(D–L) demonstrates the histopathological examination of the kidney in each experimental group. A normal histological picture was detected in the sham group (D, E). The kidney of the IR group was characterized by tubular injury which was manifested by tubular dilation, loss of brush border, tubular degeneration, hyaline casts, and mononuclear cell infiltration (F–J). The V + IR group showed improved histopathological findings (K, L).

### Effect on oxidative and nitrosative stress markers

**Figure 2.**
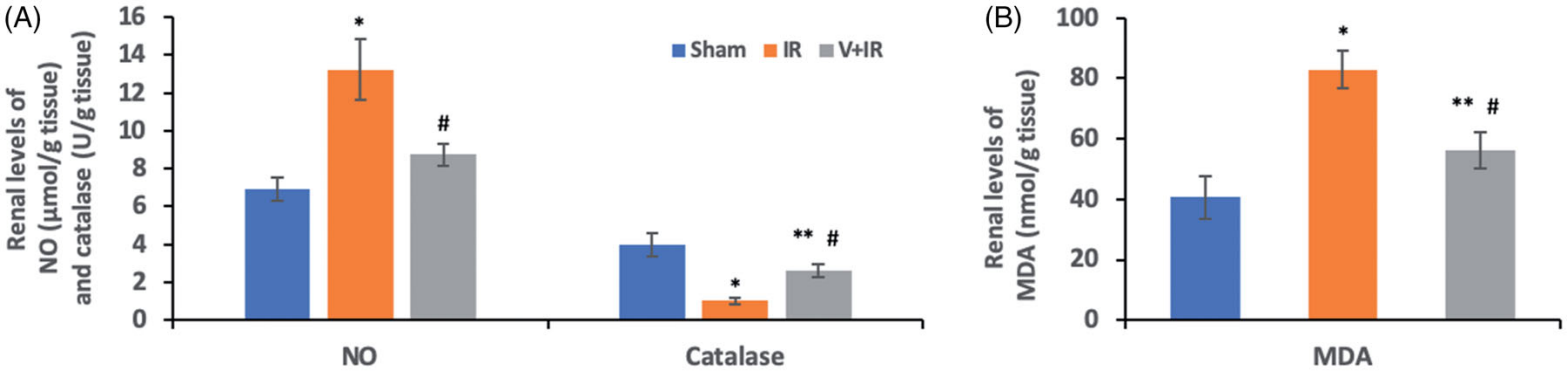
Effect of liver ischemia/reperfusion (IR) alone or in combination with vildagliptin (V, 10 mg/kg, intraperitoneally) administration for 10 days on the renal tissue contents of (A) nitric oxide (NO), catalase, and (B) malondialdehyde (MDA). Data were expressed as mean ± SD. *p<.001 compared to sham group. **p<.05 compared to sham group. ^#^*p*<.001 compared to IR group.

Oxidative and nitrosative stress were assessed as shown in Figure 2 by determining catalase (A) and MDA (B) in addition to NO (A), respectively, in renal tissue homogenate. A marked elevation in NO (by 90.5%) and MDA (by 104%) levels with a marked decrease in catalase levels (by 74.1%) were observed in the IR group compared to the sham group (*p*<.001).

Moreover, vildagliptin pretreatment in the V + IR group showed a remarkable decrease in renal levels of NO (by 33.9%) and MDA (by 32.3%) with a marked rise in catalase renal levels (by 1.5 folds) in comparison to IR group (*p*<.001).

### Effect on inflammation

A significant rise in renal TNF-α protein level was detected in the IR group when compared with the sham group as illustrated in Table 3 while, vildagliptin pretreated rats exhibited a significant reduction in renal TNF-α protein level in comparison to IR rats (*p*<.001).

**Table 3.** Effect of liver ischemia/reperfusion (IR) alone or in combination with vildagliptin (V, 10 mg/kg, intraperitoneally) administration for 10 days on the renal tissue levels of tumor necrosis factor-alpha (TNF-α).

Group	Sham	IR	V + IR
TNF-α (pg/ml)	1152 ± 117.6	2631.6 ± 137.5*	1571.8 ± 151.9**^,^^#^

Results were expressed as mean ± SD.

^*^ *p*<.001 compared to sham group.

^**^ *p*<.05 compared to sham group.

^#^ *p*<.001 compared to IR group.

### Effect on TGF-β, smad2, and smad3 mRNA expressions

**Figure 3.**
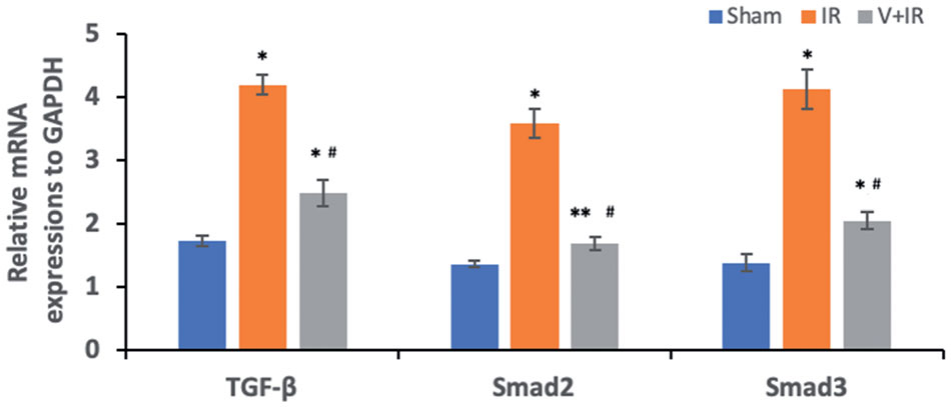
Effect of liver ischemia/reperfusion (IR) alone or in combination with vildagliptin (V, 10 mg/kg, intraperitoneally) administration for 10 days on the renal mRNA expressions, of transforming growth factor-β (TGF-β), Smad2, and Smad3. Data were presented as mean ± SD. *p<.001 compared to sham group. **p<.05 compared to sham group. ^#^*p*<.001 compared to IR group.

Figure 3 represents the determination of TGF-β, smad2, and smad3 mRNA expressions in renal tissues of all studied groups. Hepatic I/R induced a significant upregulation of renal mRNA expressions of TGF-β, smad2, and smad3 by 1.4, 1.6, and 2 folds respectively when compared with the sham group. The V + IR group exhibited a marked downregulation of renal mRNA expressions of TGF-β, smad2, and smad3 by 40.9%, 53.2%, and 50.4% respectively when compared with the IR group (*p*<.001).

### Effect on α-SMA mRNA expression

**Figure 4.**
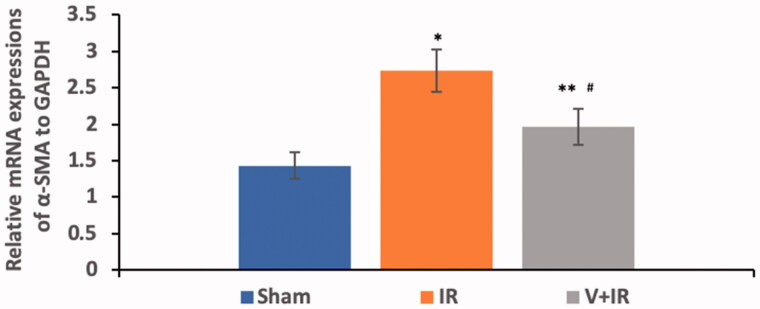
Effect of liver ischemia/reperfusion (IR) alone or in combination with vildagliptin (V, 10 mg/kg, intraperitoneally) administration for 10 days on the renal mRNA expression of α-smooth muscle actin (α-SMA). Data were presented as mean ± SD. *p<.001 compared to sham group. **p<.05 compared to sham group. ^#^*p*<.001 compared to IR group.

Hepatic I/R induced a significant upregulation in renal mRNA expression of α-SMA by 90.9% when compared with the sham group while pretreatment with vildagliptin showed a marked downregulation of renal mRNA expression of α-SMA by 28.2% in comparison to IR group as demonstrated in Figure 4 (p<.001).

## Discussion

Kidney injury is common following liver I/R injury with increased mortality and morbidity among patients [5]. Our study documented a marked elevation in serum levels of AST and ALT after liver I/R reflecting hepatocyte damage with a significant deterioration in renal function as proved by a marked rise in serum Cr and BUN in the IR group in comparison to the sham group. Morphological alterations in the liver and kidneys following liver I/R injury were also reported including degeneration in hepatocytes, congestion, and necrosis in the liver and degeneration in the renal tubular epithelium, tubular dilatation with loss of brush border, congestion, leukocyte infiltration, hyaline cast in the kidney. Similar findings were observed in previous studies [5,11,14–17] suggesting that hepatic I/R injury damages both the morphology and the function of the kidney [16,18,19].

Moreover, several studies reported pathological alterations in kidneys according to the period of reperfusion ranging from 1 to 24 h. After 1-h kidney showed moderate hemorrhage and congestion [20], eosinophilic appearance and perinuclear vacuolic formation in tubular cells [17] after 8 h showing loss of brush border and tubular epithelium with hyaline casts and congestion [21], while other researches showed tubular dilatation and necrosis, cellular swelling, and granular casts after 24-h reperfusion [5,14,22]. Kadkhodaee et al. [23] showed that hepatic and renal dysfunction were seen markedly after 4 h than 24-h reperfusion.

Hypoxia and reoxygenation are considered the two major contributing factors for tissue damage during the period of I/R [24]. After reperfusion, ROS and proinflammatory cytokines released from activated polymorphonuclear neutrophils (PMNs) [3,24] which activated the leukocyte infiltration into the kidney leading to more release of ROS and cytokines causing oxidative damage and consequently direct kidney injury [16].

A growing body of evidence showed that the oxidative stress and NO caused endothelial dysfunction and oxidative renal damage that occurred following liver I/R injury [3,4]. Moreover, it was reported that the antioxidant enzyme catalase played a protective role versus I/R induced cell injury via scavenging the ROS with converting hydrogen peroxide into water and oxygen [20].

Our study showed a marked rise in renal MDA and NO contents with a marked reduction in renal catalase levels in the IR group in comparison to the sham group. Previous studies showed similar findings reporting a marked elevation in the lipid peroxidation marker MDA and a significant reduction in catalase levels in the kidney tissue homogenate after hepatic I/R [3,16,20].

Moreover, it was found that following hepatic I/R, there were inflammatory changes occurred in the kidney causing PMNs infiltration and upregulation of renal proinflammatory mRNA expression such as TNF-α [22,23] and this coincided with our results that showed a marked elevation of renal TNF-α content in the IR group in addition to the leukocytes cells infiltration in renal tissues following hepatic I/R.

Our previous work with a model of hepatic IR, showed a marked amelioration in liver enzymes levels, as well as liver histopathology, in vildagliptin treated group compared to sham group indicating the hepatoprotective effect of vildagliptin [11] and this finding is consistent with our current study. Moreover, vildagliptin in our study significantly ameliorated the renal function and the pathological kidney changes and reduced the oxidative stress released and inflammation following hepatic I/R.

Similarly, vildagliptin or sitagliptin showed a renoprotective effect against renal I/R injury in rats via significant reduction of oxidative stress and inflammatory markers confirming their antioxidant and anti-inflammatory effects [10,25,26]. Furthermore, it was documented that the vildagliptin has cardio- and neuroprotective effect in models of myocardial and cerebral I/R, respectively, due to its ability to reduce the oxidative stress markedly proving its antioxidant activity [27,28]. This study documented for the first time the vildagliptin renoprotective effect in renal injury following hepatic IR through suppression of oxidative stress and inflammation.

Previous studies suggested that the Smad family could act as signal integrators among inflammatory or fibrogenic pathways mediating tissue inflammation [29,30]. On the other hand, a pleiotropic cytokine TGF-β was considered as the main mediator in the development of different pathological disorders including fibrosis, angiogenesis, immunosuppression, post-trauma repair, and inflammation and its effect was mediated by the Smad 2 and Smad 3 signaling molecules [31–33]. These molecules were activated in different animal models of renal diseases as obstructive kidney diseases, nephrectomy and diabetic nephropathy [29,30,34]. Moreover, it was documented that binding of TGF-β with its receptor formed a complex with kinase activity which further phosphorylated smad2/3 and then translocated into the nucleus leading to gene expression [35].

No previous studies demonstrated the involvement of TGF-β and its downstream signaling Smads in renal injury following the hepatic ischemia. Our work showed for the first time a marked upregulation of mRNA expression of smad2, smad3, TGF-β in renal tissue following hepatic I/R when compared with sham. Our findings were in harmony with other recent studies that determined the involvement of TGF-β/smad2/3 pathway in the vascular endothelial cells after lower limb I/R [31] and also in the brain after cerebral I/R [29,33,35].

The existence of a link between the TGF-β/smad2/3 signaling pathway and inflammation was reported by a recent study that observed an activation of the TGF-β/smad2/3 signaling in a mouse model of bronchial asthma with inflammatory reaction confirming that this pathway plays role in the inflammation [36]. The TGF-β was considered as a potent inflammatory mediator for different immune cell types like neutrophils and other PMN cells [37] and its release was documented after the renal IR process [38]. The activation of the infiltrated inflammatory cells after tissue injury produced ROS and induced fibrogenic cytokines if inflammation persists and not resolved [39].

The α-SMA is a protein of the smooth muscle cells and its expression reflects the injury that occurred during periods of ischemia/or reperfusion [40]. Upregulation of α-SMA expression was seen in different experimental models including uterine and renal I/R injury [41,42]. Besides, α-SMA was reported as an early marker of kidney damage and renal dysfunction [43,44]. Similarly, our study exhibited a marked upregulation in renal α-SMA mRNA expression in the IR group.

Till now, the effect of vildagliptin in renal injury following hepatic I/R was not investigated. This study showed that vildagliptin exhibited a marked downregulation of renal TGF-β/Smad/α-SMA mRNA expression when compared with the IR group. Vildagliptin attenuated renal injury in streptozotocin-induced diabetic rats through the reduction of the increased levels of renal TGF-β induced by hyperglycemia [45]. Recently, sitagliptin blocked the TGF-β/Smad pathway leading to amelioration of diabetic nephropathy [30]. Also, alogliptin exerted a renoprotective effect in nondiabetic mice with unilateral ureteral obstruction through downstream the pathway of TGF-β/Smad/α-SMA [46].

The renoprotective effect of the DPP-4 inhibitors including vildagliptin may be attributed to the increased level of GLP-1 resulting in its antioxidant, anti-inflammatory, antifibrotic, and antiapoptotic effects [8,26,28,45,46]. The elevated levels of GLP-1 and its receptor GLP-1R were reported as a protective action of vildagliptin in models of diabetic nephropathy [45] and myocardial infarction [47]. However, it was reported that vildagliptin ameliorated the lung injury in a model of pulmonary fibrosis through a mechanism independent of GLP-1 [12]; therefore, further studies are encouraged to explore this action of vildagliptin in the model of renal injury following hepatic I/R.

The limitations of our study included: the use of a single dose of vildagliptin and this was based on the fact that this dose showed protective effect in previous studies [10,28,48]; and a group with vildagliptin alone had not been done as the safety of vildagliptin was reported in healthy subjects in both experimental and clinical studies [48,49].

## Conclusions

The upregulation of renal TGF-β/Smad/α-SMA mRNA expressions is involved for the first time in the pathogenesis of the renal injury induced following hepatic I/R injury. Vildagliptin ameliorated the remote renal injury that occurred after hepatic I/R injury through multiple actions including: reduction of the oxidative stress, and the pro-inflammatory cytokine TNF-α, in addition to blocking the upregulation of TGF-β/Smad/α-SMA mRNA expressions in renal tissue.
